# Differentiation therapy for myeloid malignancies: beyond cytotoxicity

**DOI:** 10.1038/s41408-021-00584-3

**Published:** 2021-12-04

**Authors:** Ryan J. Stubbins, Aly Karsan

**Affiliations:** 1 Michael Smith Genome Sciences Centre, BC Cancer Research Institute, Vancouver, BC Canada; 2 Leukemia/BMT Program of BC, BC Cancer, Vancouver, BC Canada; 3 Department of Pathology and Laboratory Medicine, University of British Columbia, Vancouver, BC Canada

**Keywords:** Myelodysplastic syndrome, Acute myeloid leukaemia, Differentiation

## Abstract

Blocked cellular differentiation is a central pathologic feature of the myeloid malignancies, myelodysplastic syndrome (MDS) and acute myeloid leukemia (AML). Treatment regimens promoting differentiation have resulted in incredible cure rates in certain AML subtypes, such as acute promyelocytic leukemia. Over the past several years, we have seen many new therapies for MDS/AML enter clinical practice, including epigenetic therapies (e.g., 5-azacitidine), isocitrate dehydrogenase (IDH) inhibitors, fms-like kinase 3 (FLT3) inhibitors, and lenalidomide for deletion 5q (del5q) MDS. Despite not being developed with the intent of manipulating differentiation, induction of differentiation is a major mechanism by which several of these novel agents function. In this review, we examine the new therapeutic landscape for these diseases, focusing on the role of hematopoietic differentiation and the impact of inflammation and aging. We review how current therapies in MDS/AML promote differentiation as a part of their therapeutic effect, and the cellular mechanisms by which this occurs. We then outline potential novel avenues to achieve differentiation in the myeloid malignancies for therapeutic purposes. This emerging body of knowledge about the importance of relieving differentiation blockade with anti-neoplastic therapies is important to understand how current novel agents function and may open avenues to developing new treatments that explicitly target cellular differentiation. Moving beyond cytotoxic agents has the potential to open new and unexpected avenues in the treatment of myeloid malignancies, hopefully providing more efficacy with reduced toxicity.

## Introduction

Myelodysplastic syndrome (MDS) and acute myeloid leukemia (AML), the two most common myeloid malignancies, have recently seen a wave of novel therapeutics approved. While the outcomes for MDS/AML have historically been poor, this is now starting to change [1]. Initial advances in MDS/AML therapy came with the development of cytotoxic chemotherapy and allogeneic stem cell transplant [2, 3]. This was followed almost three decades later by the approval of hypomethylating agents (HMAs), such as 5-azacitidine (5AZA), for the treatment of MDS/AML [4]. During this time lenalidomide also emerged as a therapy specific for MDS with a deletion of the long arm of the 5q chromosome (del5q) [5]. Subsequently, we have seen the development of several novel therapies, including those targeting mutations in the isocitrate dehydrogenase (IDH) enzymes [6] and fms-like kinase 3 (FLT3) [7, 8], as well as combination therapies such as azacitidine with the B-cell lymphoma 2 (BCL2) inhibitor venetoclax [9]. While these agents were not necessarily developed with the intent of manipulating cellular differentiation, new data suggest that a central part of the mechanism for several of these drugs (e.g., IDH inhibitors) is inducing cellular differentiation, with subsequent apoptosis of the differentiated malignant cells [10].

Preclinical drug development in myeloid malignancies, as in other malignancies, has historically focused on differential cytotoxicity as the major goal. While this approach has led to significant advances, a focus on cytotoxicity may now be delivering diminishing returns. Myeloid malignancies are unique in oncology as treatment protocols focused on inducing cellular differentiation already exist. The most striking example is the case of acute promyelocytic leukemia (APL); the development of the all-trans retinoic acid (ATRA) and arsenic trioxide (ATO) regimen (the “Lo-coco regimen”) in 2013, notable for consisting of two drugs that lack significant intrinsic cytotoxicity, has led to complete remission (CR) and overall survival (OS) rates of greater than 95% [11]. This suggests that targeting cellular differentiation programs may be a fruitful area to be further explored in both myeloid malignancies and other cancers. Theoretical benefits of differentiation therapies may be fewer systemic side effects and lower propensity for clonal selection with subsequent resistance development, as observed with the low relapse rates generated by the Lo-coco regimen.

In this review, we outline the major scientific and clinical milestones in differentiation therapy, and suggest that these approaches may open avenues beyond cytotoxicity for the treatment of myeloid malignancies, ultimately producing new agents with high efficacy and reduced toxicity.

### Recent advances in understanding hematopoietic differentiation

Hematopoiesis remains one of the best characterized differentiation pathways, and one in which differentiation blockade is known to be a prominent mechanism of malignant transformation. This has historically been modeled as a hematopoietic differentiation “cascade” with distinct, non-overlapping intermediate cell states, defined by immunophenotype, existing between stem/progenitor and terminally differentiated cells [12–15]. This model has taken the field far, but it is now clear that hematopoietic differentiation is more complex and less discrete than this model would suggest. Partially informed by single-cell studies, it is now appreciated that immunophenotypically defined hematopoietic cell populations are intrinsically heterogeneous, and that epigenomic signatures primarily define distinct functional cell states. Farlik et al. mapped the DNA methylation profiles of single immunophenotypically defined hematopoietic stem cells (HSC) [16]. Single-cell whole genome bisulfite sequencing (scWGBS) identified distinct methylation profiles in HSC as well as multipotent (MPP), myeloid, and lymphoid progenitors [16]. They demonstrated that unique methylation signatures define hematopoietic differentiation states, with different degrees [17] of genome-wide methylation and unique patterns of targeted hypomethylation at cell-type specific TF binding sites [16, 17]. There is also an interdependent role for alterations to 3-dimensional (3D) chromatin conformation in hematopoietic differentiation. Transposon-accessible chromatin sequencing (ATAC-seq) has demonstrated that CCTC-binding factor (CTCF) sites, which are often sensitive to methylation status, are engaged in short-term HSC, repressing a host of quiescence pathways [18, 19, 21]; CTCF binding has also been shown to be aberrant in AML, with enrichment of CTCF binding at motifs for key myeloid transcription factors such as *CEBPA*, *PU.1*, and *RUNX1* [20]. These findings suggest that a renewed focus on discovering agents that can manipulate DNA methylation, chromatin conformation, and TF binding will likely be the most fruitful territory to explore in the search for novel differentiation-inducing drugs.

Clonal heterogeneity extends to the malignant state [21]. AML is an oligoclonal disease with a mix of different mutational profiles in individual clonal populations [22, 23]. Frequently, somatic mutations in epigenetic regulatory genes are shared between clones, with activating mutations (e.g., *FLT3*) often being found in a single subclonal population. This clonal composition shifts in response to cytotoxic therapy, ultimately resulting in disease relapse. Indeed, a commonly observed pattern is that cytotoxic therapy will eradicate clonal populations harboring activating mutations (e.g., *FLT3*) and achieve a morphologic remission, though persistence of low-level clones harboring epigenetic regulator mutations (e.g*.*, *TET2*, *DNMT3a*) can be observed [24]. In some studies, persistence of these clones has been linked to a higher risk of relapse in absence of allogeneic stem cell transplantation, though some studies have demonstrated conflicting results around their prognostic relevance [24–26]. Dysregulation of critical epigenomic states is a common mechanism of differentiation blockade in MDS/AML. One potential benefit of targeting differentiation is that it may avoid this well-documented phenomenon of clonal selection, which plays a prominent role in disease relapse in MDS/AML. Rather than targeting the rapidly dividing subclone while leaving a residue of slower growing malignant cells with a differentiation block and an aberrant epigenomic profile, drugs which enforce terminal differentiation programs may be able to target a broader range of clonal populations.

### Inflammation, the marrow microenvironment, and differentiation

**Fig. 1:**
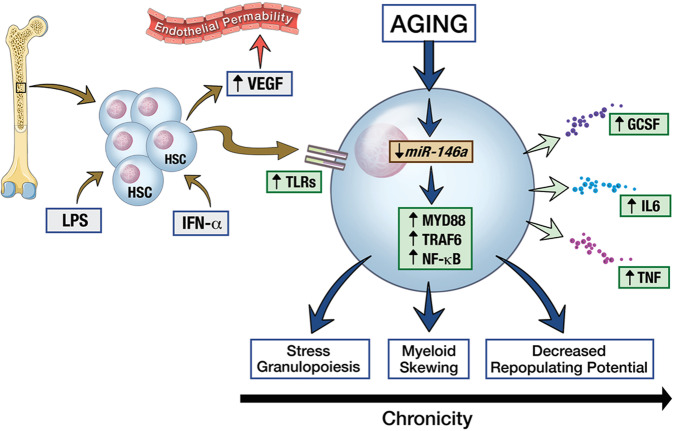
The role of inflammation in hematopoietic stem cell (HSC) differentiation—Inflammation and antigen stimulation have a number of downstream effects on HSC differentiation. Extrinsic stimuli, such as interferon alpha (IFN-α) or lipopolysaccharide (LPS) have been shown to activate toll-like receptors (TLRs) 2, 4, and 9 on HSCs. This can result in secretion of vascular endothelial growth factor (VEGF), which increases endothelial permeability. It also results in intracellular activation of MYD88, leading to downstream activation of TRAF6 and NF-kB. Similarly, aging results in a decrease in *miR-146a* expression, which can also activate MYD88, TRAF6, and NF-kB. This activation results in autocrine and paracrine signaling through cytokines and chemokines, such as granulocyte colony stimulating factor (G-CSF), interleukin 6 (IL6), and tumor necrosis factor (TNF). In the acute state this results in stress granulopoiesis and terminal myeloid differentiation. Over time, prolonged signaling can result in myeloid skewing and a loss of HSC repopulation potential.

Characteristics that affect hematopoietic differentiation extend beyond those intrinsic to the HSCs. Specifically, there is emerging evidence for the role that inflammation and aging (“inflammaging”) can play in driving both normal and aberrant differentiation (Fig. 1). There are two primary mechanisms by which HSCs are affected by inflammation: extrinsic stimulation through chemokine and cytokine signaling, and intrinsic mechanisms downstream of direct pathogen recognition. With regards to cytokine-driven pathways, previous studies used interferon-α (IFN-α) to provoke an acute inflammatory response in mice, which was found to lead to a proliferation of endothelial cells in the bone marrow, partially mediated by vascular endothelial growth factor (VEGF) secretion from bone marrow cells and HSCs [27]. This was found to lead to increased vascular permeability and the release of immune cells from the marrow [27]. Pathogenic stimuli (either lipopolysaccharide or *Escherichia coli*) have also been shown to enforce granulopoiesis and differentiation through activation of the toll-like receptors (TLRs), as well as signaling through myeloid differentiation protein 88 (MYD88) and, downstream, granulocyte colony stimulating factor (G-CSF) [28, 29]. TLRs are predominantly responsible for intrinsic signaling and direct mechanisms that drive progenitor differentiation down a myeloid pathway [30–33]. Cytokine stimulation and TLR signaling are not independent, however. HSCs are known to engage in paracrine signaling, with a significant amount of cytokine secretion after TLR stimulation, and this has been shown to be an important mechanism behind stress granulopoiesis [34]. Chronic inflammation is also known to imprint myeloid bias into HSCs through trained immunity. The administration of β-glucan has been shown to result in the expansion of myeloid-biased progenitors, and progenitor cells previously exposed to β-glucan retain an increased myeloid potential upon rechallenge, demonstrating that inflammatory stimuli can permanently imprint the latter differentiation programs of HSCs [35]. Similarly, it is known that myeloid skewing in autoimmune arthritis occurs at the level of the HSC, showing that previous exposure to inflammatory stimuli can permanently perturb the downstream differentiation programs of HSCs [36]. Thus, inflammation has a physiologic role to play in driving differentiation of both HSCs and committed progenitors and enforcing a myeloid differentiation program in response to infections or other stress stimuli. (e.g., “stress granulopoiesis”). This occurs through a mix of both cytokine-mediated and intrinsic signaling pathways, with the TLRs playing a central role.

Inflammation and immune activation, and the associated impacts on cellular differentiation, also have relevance in MDS. Several immune signaling related proteins have been shown to be aberrantly expressed in MDS, including TLRs [37] as well as downstream effectors such as *MYD88* [38], *IRAK1* [39] and the ubiquitin ligase *TRAF6* [40]. Intrinsic regulators of immune signaling, such as *miR-145* and *miR-146a*, have also been shown to be downregulated in MDS [41]. These changes seen in established MDS may have their roots in antecedent inflammation-driven myeloid-skewing and HSC dysfunction. It has been shown that IL-1β can drive HSC proliferation [42], induce myeloid differentiation via Batf2 and C/EBPβ [43, 44], and promote megakaryopoiesis in primed (CD41^hi^) HSCs [45]. Alterations to intrinsic regulators of aging-associated inflammation have been shown to drive HSC dysfunction and predispose to myeloid malignancy. Grants et al. showed that *miR-146a* expression declined in older mice, and that *miR-146a* null young mice demonstrated premature HSC aging by depleting quiescent HSCs through IL6 and tumor necrosis factor (TNF) activation, predisposing these mice to myeloid malignancy [46]. Similarly, it has been demonstrated that loss of *miR-143/145* depletes functional HSCs through the transforming growth factor β (TGFβ) pathway [47]. However, despite the loss of normal HSCs, many of the mice from this study developed a leukocytosis and a transplantable myeloid malignancy due to the expansion of a transformed, malignant progenitor population [47]. The effects of inflammation are an important consideration in hematopoietic differentiation status, particularly in the context of MDS/AML, and may be a target for future approaches that modify differentiation status.

### Linking differentiation to therapy

As we have outlined, the modern view of hematopoietic differentiation is more nuanced than historically appreciated. Physiologic hematopoietic differentiation is largely epigenetically regulated and characterized by a pool of precursors that become lineage-restricted in a dynamic and flexible fashion. It is also clear that the traditional immunophenotypically defined cell compartments are intrinsically heterogeneous, both on genomic and functional bases. Alterations to regulators of normal hematopoietic differentiation also seem to be a key feature in the development of myeloid malignancies. This may relate to a failure of intrinsic regulators of normal differentiation (e.g., somatic mutations in epigenetic regulators) or chronic external stimuli (e.g., inflammation) that promote the development of subsequent clonal disorders. Despite the importance of abnormal differentiation to pathogenesis in MDS/AML, explicitly targeting these pathways has not been a focus in drug development for these diseases. However, as we outline below, induction of differentiation is an important part of the mechanism of action of several successful therapeutic approaches. While there have not been specific studies directly examining the impact of inflammation on therapeutic outcomes, we do know that inflammation is strongly correlated with several secondary features that result in inferior therapy outcomes (e.g., advanced age, comorbidities, poor functional status). There is significant interest in integrating measures of the “immunome” into future clinical trials [48]. Characterizing the interaction between therapy resistance, systemic inflammation, and differentiation blockade should be prioritized as a correlative when trying to understand the efficacy of novel therapies.

## The original differentiation therapy: all-trans retinoic acid and arsenic in acute promyelocytic leukemia

The prototypical malignant cell differentiation therapy is the ATO and ATRA combination for APL. APL is characterized by a fulminant clinical presentation with bleeding and thrombosis and usually carries the classical translocation of chromosome 15 and 17 t(15;17) [49, 50]. The consequence of this translocation is the fusion of the promyelocytic leukemia (*PML*) gene with the retinoic acid receptor-alpha (*RARα*) gene to form the *PML-RARα* fusion transcript; this results in a myeloid differentiation block at the promyelocytic stage [49]. ATRA binds the retinoic acid receptor component of the transgene, displacing bound corepressor complexes and promoting degradation of the PML-RARα fusion protein [51]. ATO acts by blocking the transcriptional repression function of the PML-RARα fusion protein by triggering phosphorylation of SMRT through the mitogen-activated protein kinase (MAPK) pathway. This results in a relocalization of SMRT from the nucleus to the cytoplasm [52]. The downstream effect of this combination removes the transcriptional repression induced by the fusion protein, and effectively removes the differentiation blockade that is in place. This therapy-induced differentiation results in hyperleukocytosis with a characteristic differential profile that is comprised of a mix of mature myeloid cells with an eventual clearance of the malignant blasts [53]. The treatment course is generally long for the ATO-ATRA combination, at 32 weeks in the original Lo-coco trial. A longer treatment course with gradually accruing remissions is a consistent feature of other therapies involving differentiation as a mechanism of action also [4, 54, 55].

It was quickly recognized with the ATO-ATRA combination that some patients would develop a syndrome characterized by the acute onset of fevers, volume overload, and pulmonary edema, which was dubbed “differentiation syndrome” (DS) [53]. DS has also subsequently been observed in the context of other novel therapies such as the IDH inhibitors. The specific mechanism of DS in APL is thought to be related to chemokine secretion after the malignant promyelocytes are exposed to ATO and ATRA. One study used an in vitro model to identify that the stimulation of APL cells by ATO and ATRA caused induced expression of CC-chemokines [56]. Another study examined the role of CXC chemokines (MIP-2 and KC) along with *ICAM-1* in a murine model of DS. They found that ATRA treatment increased gene expression of the CXC chemokines and *ICAM-1* in the lung alveolar cells, and *MIP-2* was observed on the alveolar macrophages [57]. Overall, the mechanism of action of DS seems to be driven by ATRA-induced expression of the CC- and CXC-chemokine families resulting in neutrophil and monocyte recruitment to affected tissues that is possibly compounded by increased expression of adhesion molecules such as *ICAM-1*. Outside of the context of ATRA therapy, it may be that DS is primarily driven by chemokine secretion from the differentiating myeloblasts, though this has largely not been explored.

There are several lessons that can be abstracted from the ATO-ATRA story in APL. The first is the striking therapeutic efficacy that this combination achieves, while provoking minimal cytotoxicity. Given that most current drug-discovery platforms utilize rapid cell death as an endpoint to assess the efficacy of compounds, neither of these compounds may have been identified in that context. However, it is important to recognize that APL is a distinct disease state with a lower degree of genetic complexity versus non-APL MDS/AML; the more pronounced clonal and genetic diversity in non-APL MDS/AML adds an additional layer of complexity to consider when examining the role for differentiation induction in therapy. In addition, ATO/ATRA directly targets the founding genetic event in APL within the leukemia stem cells, which is more difficult to achieve in non-APL leukemia stem cells. The utility of ATRA without ATO has been examined in non-APL AML. One in vitro study suggested that ATRA might be efficacious at inducing differentiation in AML with low expression of the transcriptional regulator *MN1* [58], or in the presence of mutated *NPM1* [59, 60]. Two previous studies demonstrated that ATO and/or ATRA could induce proteasome-mediated degradation of mutant *NPM1* in AML cell lines and primary samples, leading to differentiation and apoptosis [59, 60]. Other recent data suggests that non-APL AML cells overexpressing *EVI-1* may be sensitive to ATRA. Two studies demonstrated that ATRA could induce differentiation and apoptosis in *EVI-1* overexpressing non-APL cell lines and primary samples [61, 62]. Despite these encouraging preclinical findings, the role for ATRA or ATO for non-APL AML in a clinical context remains unclear [63, 64]. Other selective *RARα* agonists, such as tamibarotene (SY-1425) have been shown to induce differentiation and apoptosis in high *RARα* expressing AML primary cells [65]. Tamibarotene is in early phase clinical trials for both relapsed/refractory and unfit AML patients with high *RARα* expression and has shown encouraging results so far [66, 67].

## Epigenetic therapy and differentiation

Another type of therapy for which differentiation is increasingly recognized as a component of its mechanism are the HMAs, 5AZA and decitabine (DEC). While hypomethylation is the most direct and obvious effect of HMAs, the downstream mechanism of action is complex and likely dose-dependent, and the connections between hypomethylation and eventual blast clearance in the marrow remain to be fully defined [68]. It is interesting to note it often takes 4 or greater months of therapy to see a response with HMAs, similar to other differentiation-inducing agents. At high doses (~20 µM), a 5AZA metabolite is incorporated into cellular RNA directly, resulting in inhibition of protein synthesis and subsequent cell death [69]. However, these concentrations are not reflective of those achieved in vivo, with a pharmacologic *C*_max_ of 5AZA corresponding to approximately 4 µM, suggesting this may not be the primary mechanism by which 5AZA works in patients [70]. With regards to mechanisms at more physiologic concentrations, there has been an extensive amount of investigation into the possible role of endogenous retroelement (ERE) reactivation and the associated stimulation of an anti-leukemic response from the resident T- and natural killer (NK)-cell populations [71–73]. There has also been a competing argument, historically, suggesting that 5AZA partially works by inducing terminal differentiation, with subsequent apoptosis, in leukemic cells [74]. This has been reinforced by a recent study that used RNA sequencing (RNA-seq) to assess a cohort of MDS and chronic myelomonocytic leukemia (CMML) patients who were either primary responders or non-responders to 5AZA [75]. They comprehensively annotated ERE-related transcripts and noted that, while their expression did increase after 5AZA therapy, this did not correlate with response. Instead, they identified differential expression of developmentally regulated transcriptional signatures of both protein-coding and noncoding genes between 5AZA responders and non-responders [75]. This suggests that a major component of the 5AZA mechanism of action may be through activating latent differentiation programs in the malignant blasts, and that the induction of ERE transcripts may be a bystander effect, though this remains to be fully explored. Reports of clinical DS with 5AZA treatment have been noted, but this is rare [76]. Both 5AZA and DEC irreversibly bind a broad range of DNA methyltransferases, partially accounting for their toxicity profile. Novel competitive inhibitors that are selective for DNMT1 (e.g., GSK3685032) have been developed, and may produce fewer hematologic toxicities than conventional HMAs [77]. Recently, there has also been emerging data about the association between ten-eleven translocation 2 (*TET2*) and *IDH* mutations, ascorbic acid (Vitamin C), and the epigenome, as vitamin C has been shown to stimulate TET2 catalytic activity in vitro [78]. In one study using a murine model of *IDH1* mutated AML, vitamin C exposure induced a pattern of differentially methylated regions that overlapped with enhancers implicated in myeloid differentiation, specifically regions related to the hematopoietic-specific TFs: CEBPβ, HIF1α, RUNX1, and PU.1. Chromatin immunoprecipitation sequencing confirmed a loss of PU.1 and increase in RUNX1-bound elements and an increase in H3K27ac flanking sites near RUNX1-bound sequences [78]. The authors suggest that vitamin C may have a role in inducing epigenetic remodeling of differentiation-related TF binding sites in this AML model. It has also been suggested that vitamin C may be synergistic with DEC in promoting activation of TET2 [79], though this area requires further investigation.

There are several other epigenetic therapies currently in clinical trials for MDS and AML (Table 1). Specifically, a number of epigenetically targeted agents have recently been developed for leukemia patients that harbor the *MLL* fusion protein, which comprise about 10% of acute leukemias. The *MLL*-rearranged fusion protein binds to and dysregulates proliferation-associated genes such as the *HOXA* cluster, *MEIS1*, and *PBX3*. However, both native and rearranged *MLL* must bind with a chromatin-associated protein complex, which includes members such as disruptor of telomeric silencing 1-like (DOT1L) and Menin [80]. Two groups have developed small molecules that inhibit the *MLL-*Menin interaction, which have demonstrated efficacy in a patient-derived xenograft model of *MLL*-rearranged leukemia. This approach is being tested in early phase clinical trials [80, 81]. There is also evolving data for the use of Menin inhibitors in AML harboring *NPM1*-mutations [82, 83] or *NUP98* rearrangements [84]. There have also been a number of DOT1L inhibitors recently developed (EPZ-5676), which function by inhibiting the DOT1L enzyme and thus relieving the inappropriate methylation of H3K79 [85]. *HOXA9* overexpression is thought to be a downstream transcriptional consequence of the *MLL-*rearrangement; it has been shown that inhibition of dihydroorotate dehydrogenase (DHODH) relieves the *MLL*-induced differentiation block by modulating mitochondrial metabolism [86]. Inhibitors of DHODH are currently in early phase trials (Brequinar) [87].

**Table 1:** Summary of epigenetically targeted agents.

Summary of epigenetically targetted agents actively being studied						
Target	Drug Name	Disease State	Mechanism	Differentiation	Trial Phase	NCTN/PMID
DNMT1-3	5-Azacitidine	MDS, AML	Hypomethylating	+	Phase 3	*Lancet Oncol*. (2009) 10;223–232
	Decitabine					*Cancer* (2006) 106;1794–1803
DNMT1	GSK3685032	MDS, AML	Hypomethylating	Unknown	PreClinical	*Nature Cancer* (2021)2;1002–1017
BET	CPI-0610 OTX015	MPN, MDS, AML	Blocks acetylated histones	+	Phase 1b, 2, & 3	NCT02158858 NCT04603495 NCT01713582
Menin	SNDX-5613	*MLL-*rearranged or *NPM1*-mt leukemia	Blocks Menin-*MLL* interaction	Unknown	Phase 1	NCT04065399
	JNJ-75276617			Unknown	Phase 1, 2	NCT04811560
	KO-539			Unknown	Phase 1, 2	NCT04067336
	DS-1594b	R/R AML and ALL		+++	Phase 1, 2	NCT04752163
	BMF-219	TBD		Unknown	TBD	Pending
DHODH	Brequinar	R/R AML, ALL, MPAL	Alters mitochondrial metabolism	+++	Phase 1b, 2A	NCT03760666
DOT1L	EPZ-5676	*MLL*-rearranged AML or ALL	Demethylates H3K79	+++	Phase 1	NCT01684150

*DNMT1* DNA methyltransferase 1, *DOT1L* like histone lysine methyltransferase 1, *DHODH* dihydroorotate dehydrogenase, *BET* bromodomain and extraterminal proteins, *MDS* myelodysplastic syndrome, *MPN* myeloproliferative neoplasm, *AML* acute myeloid leukemia, *ALL* acute lymphoblastic leukemia, *MPAL* mixed-phenotype acute leukemia [90].

Another epigenetic therapy in early phase trials are the bromodomain and extraterminal (BET) inhibitors (e.g., CPI-0610). Normally BET proteins, such as Bromodomain-containing protein 4 (BRD4), bind acetylated histone tails, bringing together the elongation complex and the promoter region [88, 89]. One study compared compounds in early clinical trials for several of these epigenetic regulators, including inhibitors of BET, DHODH, DOT1L, Menin, as well as a CDK9 inhibitor [90]. Using a unique in vitro model the authors examined inhibition of proliferation and apoptosis, as well as differentiation induction with phagocytosis as a functional readout [90]. Menin-MLL and DOT1L inhibitors appear to act specifically on *MLL*-rearranged cell lines, while inhibitors for BET, DHODH, and CDK also impact non-*MLL*-rearranged cells. Interestingly, they identified significant differentiation induction effects for inhibitors of Menin-MLL, DOT1L, and DHODH while the BET and CDK9 inhibitors primarily acted by apoptosis induction [90].

One other class of agents that have been investigated are inhibitors of the histone demethylase LSD1 (also known as KDM1A), which is a component of the MLL-protein complex. One preclinical study demonstrated that LSD1/KDM1A inhibition with a small molecule (ORY-1001) induced H3K4me2 accumulation on KDM1A target genes, resulting in blast differentiation and reduced leukemia burden in cell line and animal models of *MLL*-rearranged T-acute lymphoblastic leukemia [91]. These promising agents emphasize that it is important to consider not just the relevant TFs and epigenetic factors involved in malignancy associated differentiation blockade, but to also consider other mechanistically relevant aspects of TF binding and gene regulation (e.g., co-factors and metabolic enzymes) as possible targets for intervention.

## Mutationally targeted therapy and differentiation: IDH and FLT3 inhibitors

IDH and FLT3 inhibitors are rapidly being incorporated into the therapeutic algorithms for myeloid malignancies. IDH inhibitors are another example of successful agents for myeloid malignancies that work partly through differentiation induction. Clinical DS has been a relatively commonly reported adverse event at 18% for ivosidenib [92] and 7% for enasidenib [55]. Compared to the DS seen with ATO-ATRA therapy in APL, the median time to onset is significantly longer (48 vs. 11 days), though the clinical presentation and management are similar [93]. The mechanism by which the IDH inhibitors are thought to act is by suppressing production of the oncometabolite 2-hydroxyglutarate (2-HG), which is produced by the mutant enzyme [94]. Elevated levels of 2-HG induce epigenetic remodeling by inhibiting TET2 and Jumonji C domain-containing histone demethylases, which is partially responsible for the differentiation blockade seen in IDH-mutated myeloid malignancies [95, 96]. Inhibitors of mutant IDH1/2 proteins decrease the intracellular levels of 2-HG, which induces cellular differentiation, possibly through altering DNA methylation patterns, or alternatively through regulation of histone methylation [97, 98]. While there is some limited in vitro evidence [97] to support a link to DNA methylation, there are no studies confirming it in patient samples. Similar to what is seen with other therapies that manipulate differentiation, the median time to achieve a disease response is prolonged, often taking 4–6 months before a maximal response is seen [6, 92]. In addition, changes to cellular metabolism (e.g., through 2-HG induction) are linked to epigenetic changes, which in turn are related to differentiation state. Given the numerous metabolic and mitochondrial abnormalities described in AML [99], the interplay between these three factors should be examined in more detail.

Somewhat more unexpectedly, there is an evolving body of literature examining the role of cellular differentiation in the mechanism of the novel FLT3-inhibitors. FLT3-internal tandem deletions (FLT3-ITD) are classically thought of as a “driver” mutation in MDS/AML that are usually a later event in AML evolution [100]. These mutations are not typically linked with differentiation blockade and cell state in the same way that epigenetic regulator mutations or IDH mutations have been. One study examined the role of quizartinib, a second-generation FLT3-ITD inhibitor, and differentiation in primary AML patient samples [101]. They observed that in 13 of 14 FLT3-ITD AML patients treated with quizartinib that terminal myeloid differentiation of the bone marrow blasts was observed. They then demonstrated with an in vitro co-culture model with human bone marrow stroma and FLT3-ITD mutated primary blast cells that quizartinib induced terminal differentiation [101]. In patients treated with gilteritinib, clinical DS has been reported in up to 3% of patients [7]. Another study examined differentiation induction in primary samples from AML patients treated with gilteritinib [102]. They demonstrated a differentiation response in 10 of 21 bone marrow samples in response to gilteritinib, and this was associated with a reduction in the malignant blast fraction in the marrow. Notably, this is higher than the observed incidence of observed DS, suggesting that the amount of differentiation being induced may often be below the threshold to manifest clinical DS. The remaining patients appeared to develop a response through an alternative, non-differentiation-related pathway. The reasons for individual heterogeneity may relate to other co-existing mutations and the epigenetic cell state, though this has yet to be explored. These authors also demonstrated evidence of persistent clonal hematopoiesis in patients in whom differentiation was induced, suggesting that the nature of the response achieved differs fundamentally from that seen with cytotoxic agents [102]. These data suggest that the clinical efficacy of FLT3 inhibition is, in part, mediated by induction of cellular differentiation, re-emphasizing the importance of differentiation to effective therapy in myeloid malignancies. It also emphasizes that the relative roles and importance of differentiation and cytotoxicity may be contextually dependent upon the distinct set of co-existing mutations and other unexplored factors.

## The role of megakaryocytic differentiation: dasatinib and lenalidomide

Historically, megakaryocyte differentiation has been viewed as arising from a common myeloid-erythroid progenitor downstream of the multipotent progenitor (MPP) cell subpopulation. More recently, a new understanding has emerged that suggests most megakaryocytes derive directly from a megakaryocyte-biased HSC pool [103]. Megakaryocyte-biased HSCs have been shown to express lineage-specific surface markers such as von Willebrand factor (vWF) and CD41 and are responsive to thrombopoietin [104]. They also demonstrate a TF profile biased towards megakaryocyte differentiation, including TFs such as GATA1, GFI1b, GATA2, and FLI1 [104]. These megakaryocyte-biased HSCs are thought to rapidly differentiate to produce platelet responses after inflammatory or infectious stimuli [104]. Interestingly, dasatinib, a second-generation inhibitor of the BCR-ABL kinase, has been shown to promote megakaryocyte differentiation [105]. It has been observed that mice treated with dasatinib have a 30% increase in the number of megakaryocytes in the bone marrow despite developing peripheral blood thrombocytopenia. As well there is an increase in the number of polyploid (8–64N) megakaryocytes. Despite increased numbers, these megakaryocytes demonstrated defective migration in the presence of dasatinib, possibly through a loss of phosphorylation of the Src and Syk tyrosine kinases [105]. While the anatomical distribution of megakaryocytes was unchanged, dasatinib treatment abolished chemotaxis in response to stromal cell-derived factor 1α, and abolished proplatelet formation, possibly explaining dasatinib-induced thrombocytopenia [105].

**Fig. 2:**
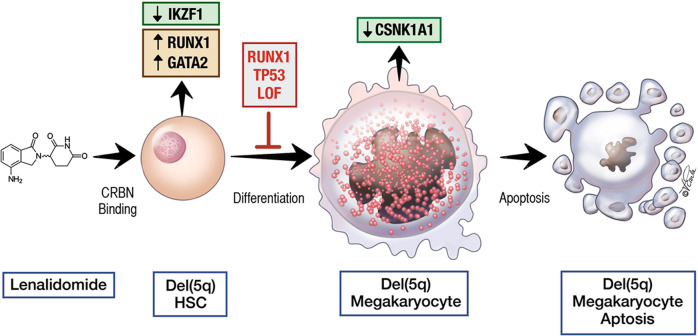
The importance of megakaryocyte differentiation to lenalidomide action in del(5q) myelodysplastic syndrome – Myelodysplastic syndrome with del(5q) is characterized by a clonal malignant del(5q) hematopoietic stem cell (HSC), which is haploinsufficient for casein kinase 1A1 (CSNK1A1). Lenalidomide binds cereblon (CRBN), which results in degradation of IKZF1 and derepression of RUNX1 and GATA2 activity, resulting in megakaryocytic differentiation. Lenalidomide binding to CRBN also promotes CSNK1A1 degradation which, in the context of haploinsufficiency, triggers megakaryocyte-selective cell death. Loss-of-function mutations in *RUNX1*, *TP53* or *GATA2* block lenalidomide-triggered megakaryocytic differentiation, and thereby apoptosis of the malignant clone.

New evidence also implicates megakaryocyte differentiation in the therapeutic response of lenalidomide in del(5q) MDS. Del(5q) is the most common cytogenetic abnormality seen in MDS, and results in haploinsufficiency for various coding and noncoding genes; this MDS subtype is uniquely sensitive to the immunomodulatory drug lenalidomide. However, at least half of the del(5q) MDS patients who respond to lenalidomide eventually become resistant. Del(5q) MDS patients who become lenalidomide resistant tend to have a either a *TP53* or *RUNX1* mutation, and in a rare case, loss of *GATA2* function (Fig. 2) [10, 106]. Ikaros, RUNX1 and GATA2 bind megakaryocyte promoters at common sites in hematopoietic cells, and lenalidomide-bound Cereblon targets Ikaros for degradation. Subsequent derepression at megakaryocyte lineage promoters allows RUNX1 and GATA2 to drive expression leading to megakaryocyte differentiation. Thus, lenalidomide activates a RUNX1/GATA2-dependent megakaryocytic program in hematopoietic cells. Although TP53 does not bind these megakaryocytic promoters, it appears to have an upstream non-transcriptional effect on megakaryocyte differentiation that is RUNX1/GATA2-dependent. Haploinsufficiency of CSNK1A1 in megakaryocytes derived from the malignant del(5q) clone makes the cells differentially sensitive to Lenalidomide/CRBN-mediated CSNK1A1 degradation and subsequent TP53-mediated apoptosis [107]. Lenalidomide/CRBN-mediated megakaryocyte differentiation is an essential requirement for subsequent apoptosis, as CSNK1A1 degradation only induces apoptosis in del(5q) megakaryocytes, but spares the erythroid and myeloid populations. In both examples, dasatinib and lenalidomide, induction of megakaryocytic differentiation is a downstream effect of these therapeutic interventions, and in the case of lenalidomide it appears central to its therapeutic activity. We also see commonalities in that both agents indirectly activate megakaryocyte specific TFs and their downstream pathways, resulting in malignant cells proceeding down a megakaryocyte differentiation pathway. As such, the evolution of mechanisms that result in de novo differentiation blockade in MDS/AML patients who are receiving active treatment may be a more common mechanism of disease relapse than previously appreciated.

## Potential and future directions

As we have outlined, aberrant cellular differentiation is a core feature of both MDS and AML. We also have seen that differentiation is a major player in the mechanism of action of many of the novel therapies being introduced in MDS and AML, and that it appears to be central to their efficacy. We have also outlined many potential benefits to exploiting differentiation induction the treatment of myeloid malignancies, such as reduced toxicity and clonal selection. One major challenge in developing therapeutics that enforce cellular differentiation is that the proteins involved are often TFs, which have not been traditionally viewed as druggable targets. However, there are creative ways to address this; in the example of lenalidomide resistance, is there a possible way to activate GATA2 in lenalidomide-resistant patients with *RUNX1* or *TP53* mutations? GATA2 is partially regulated through a positive-feedback loop wherein interleukin-1-beta and CXCL2 stimulate GATA2 expression [108]. Could interleukin-1-beta stimulation or a CXCL2 agonist overcome lenalidomide resistance by boosting GATA2 activity? Mechanisms such as this should be explored as possible ways to either overcome therapy resistance and develop new treatment approaches. We would also emphasize the importance of including differentiation endpoints in preclinical and high-throughput drug screening approaches. Overall, understanding and manipulating cellular differentiation holds promise in advancing cancer therapeutics in myeloid malignancies.
